# Tendon Extracellular Matrix Alterations in Ullrich Congenital Muscular Dystrophy

**DOI:** 10.3389/fnagi.2016.00131

**Published:** 2016-06-08

**Authors:** Francesca Sardone, Francesco Traina, Alice Bondi, Luciano Merlini, Spartaco Santi, Nadir Mario Maraldi, Cesare Faldini, Patrizia Sabatelli

**Affiliations:** ^1^Department of Biomedical Sciences, University of PadovaPadova, Italy; ^2^National Research Council of Italy, Institute of Molecular GeneticsBologna, Italy; ^3^Rizzoli Orthopaedic Institute, University of BolognaBologna, Italy; ^4^Muscle Clinic, Villa Erbosa Hospital, Gruppo San DonatoBologna, Italy; ^5^SC Laboratory of Musculoskeletal Cell Biology, IOR-IRCCSBologna, Italy

**Keywords:** Collagen VI, Ullrich congenital muscular dystrophy (UCMD), pericellular matrix, metalloproteinases, tendon

## Abstract

Collagen VI (COLVI) is a non-fibrillar collagen expressed in skeletal muscle and most connective tissues. Mutations in COLVI genes cause two major clinical forms, Bethlem myopathy and Ullrich congenital muscular dystrophy (UCMD). In addition to congenital muscle weakness, patients affected by COLVI myopathies show axial and proximal joint contractures and distal joint hypermobility, which suggest the involvement of the tendon function. We examined a peroneal tendon biopsy and tenocyte culture of a 15-year-old patient affected by UCMD with compound heterozygous *COL6A2* mutations. In patient’s tendon biopsy, we found striking morphological alterations of tendon fibrils, consisting in irregular profiles and reduced mean diameter. The organization of the pericellular matrix of tenocytes, the primary site of collagen fibril assembly, was severely affected, as determined by immunoelectron microscopy, which showed an abnormal accumulation of COLVI and altered distribution of collagen I (COLI) and fibronectin (FBN). In patient’s tenocyte culture, COLVI web formation and cell surface association were severely impaired; large aggregates of COLVI, which matched with COLI labeling, were frequently detected in the extracellular matrix. In addition, metalloproteinase MMP-2, an extracellular matrix-regulating enzyme, was increased in the conditioned medium of patient’s tenocytes, as determined by gelatin zymography and western blot. Altogether, these data indicate that COLVI deficiency may influence the organization of UCMD tendon matrix, resulting in dysfunctional fibrillogenesis. The alterations of tendon matrix may contribute to the complex pathogenesis of COLVI related myopathies.

## Introduction

Tendons are composed of relatively rare cells (tendon fibroblasts) scattered within a predominant dense connective tissue arranged in an highly ordered ECM, mainly constituted by collagen fibrils, which are hierarchically organized to withstand tensile forces transmitted from muscles to bone axis (Kadler et al., 1996). Fibrils contain mostly collagen I (COLI) and other components, which contribute to fibrillogenesis, such as collagen types III, V, VI, XII, and XIV, as well as proteoglycans and glycoproteins (Screen et al., 2015). Fibril assembly is crucial for tendon function. Fibril intermediates assemble by lateral and longitudinal association (Birk et al., 1995). The assembly of intermediates into longer, continuous fibrils with larger diameters dramatically increases the tensile strength of tissues such as the tendon. Tendon fibrillogenesis is regulated by a variety of fibril surface associated molecules, as fibronectin (FBN), decorin, biglycan and collagens types XII and XIV (Birk et al., 1995; Young et al., 2002; Zhang et al., 2005). These regulatory matrix molecules are all substrates for MMP-2/MT3-MMP, which ensures the matrix turnover required during tendon development (Jung et al., 2009).

Collagen VI (COLVI) is a microfibrillar collagen expressed in most tissues. In tendons and ligaments, COLVI forms a network of beaded filaments associated both to collagen fibrils and to the cell surface (Bruns et al., 1986; Ritty et al., 2003). The best-characterized and widely expressed form of COLVI is the [α1, α2, α3] heterotrimer that further assembles intracellularly into dimers and tetramers. After secretion, tetramers undergo end-to-end association, giving rise to the typical 100 nm-spaced beaded microfibrils (Bruns et al., 1986), which may form, alternatively, fibrils by parallel alignment, or web-like structures by multiple interconnections, depending on the association with cell receptors and ECM-binding proteins (von der Mark et al., 1984; Bruns et al., 1986; Wiberg et al., 2002; Knupp et al., 2006; Koudouna et al., 2014). In humans, two novel COLVI subunits, the α5 and α6 chains, were recently identified, which structurally resemble the α3 chain but display a more restricted and often alternative distribution pattern (Fitzgerald et al., 2008; Gara et al., 2008; Sabatelli et al., 2011, 2012). In tendons, the [α1, α2, α3] heterotrimer is abundantly expressed (Thakkar et al., 2014), whereas the α5 chain is selectively detected at the myotendinous junction and the α6 chain is absent (Sabatelli et al., 2012).

Mutations in the genes encoding COLVI (*COL6A1, COL6A2, and COL6A3*) cause the COLVI-related myopathies, which comprise two major clinical forms, Bethlem myopathy (BM [MIM 158810]) and Ullrich congenital muscular dystrophy (UCMD [MIM 254090]), and the limb girdle and the Myosclerosis myopathy (MM) variants. UCMD is a severe disorder characterized by congenital muscle weakness; BM is a mild form characterized by slowly progressive axial and proximal muscle weakness; MM is characterized by slender muscles with firm “woody” consistence and restriction of movement of many joints (Merlini and Bernardi, 2008). COLVI myopathies are also characterized by joint hyperlaxity and contractures. Type and distribution of contractures are distinguishing features of COLVI disorders. The UCMD is characterized by proximal contractures and distal laxity, BM by distal contractures, limb-girdle phenotype by late or no contractures, and MM by early, diffuse, and progressive muscle contractures resulting in severe limitation of movement of all axial, proximal, and distal joints (Merlini and Bernardi, 2008). Patients affected by COLVI myopathies may also display skin abnormalities, like keloids or “cigarette article” scars, dry skin, striae rubrae, and keratosis pilaris (follicular keratosis). Thus, COLVI mutations result in disorders with combined muscle and connective tissue involvement.

Animal models of COLVI myopathies have been developed in mice (Bonaldo et al., 1998; Pan et al., 2013) and zebrafish (Telfer et al., 2010; Zulian et al., 2014). Recently, a nonsense variant in *COL6A1*−/− has been detected in Landseer dogs (Steffen et al., 2015). *COL6A1* mice, a COLVI null model (Bonaldo et al., 1998), and *COL6A3* deficient mice (Pan et al., 2013) develop a mild myopathy and tendon dysfunction possibly due to altered tendon fibrillogenesis (Izu et al., 2011; Pan et al., 2013). *COL6A1* morphant zebrafish display a severe impairment of motor function and myotendinous junction abnormalities (Telfer et al., 2010; Zulian et al., 2014). Altogether, these data point to an involvement of COLVI in the regulation of tendon function.

In order to determine whether the altered tendon fibrillogenesis reported in animal models of COLVI myopathies is also present in humans, we studied a tendon biopsy and tenocyte cultures of a UCMD patient with compound heterozygous mutations in *COL6A2* gene (Martoni et al., 2009). We found changes consistent with altered fibrillogenesis, as indicated by fibrils abnormalities, and *in vitro* alterations of COLI organization and metalloproteinase MMP-2 activity.

## Materials and Methods

### Tendon Biopsies

Peroneal tendon biopsies were harvested from two healthy subjects (17 and 21) during foot surgery and during a Grice procedure from a previously genetically characterized UCMD patient carrying compound heterozygous mutation for a G > A variation at position +5 of *COL6A2* intron 8 and a nonsense mutation R366X in *COL6A2* exon 12 (Martoni et al., 2009). All patients granted informed consent. Tendon fragments were subjected to mechanical dissociation, and maintained in Dulbecco’s Modified Eagle Medium (DMEM) containing 1% antibiotics plus 10% Fetal Bovine Serum (FBS; Nemoto et al., 2013); 0.25 mM L-ascorbic acid was added to the medium to allow COLVI tetramer secretion (Engvall et al., 1986).

### Immunofluorescence and Confocal Analysis

The immunofluorescence analysis with anti-COLVI (Millipore) on tendon cell cultures was performed as previously reported (Sabatelli et al., 2012). Cells grown onto coverslips were incubated with antibodies against FBN (Sigma), COLVI (Millipore), COLI (Abcam), and with FITC or TRITC-conjugated anti-mouse or anti-rabbit secondary antibodies (DAKO). Cell nuclei were stained with 1 mg/ml DAPI (Sigma-Aldrich). Samples were mounted with an anti-fading reagent (Molecular Probes). The confocal imaging was performed with a Nikon A1-R confocal laser scanning microscope, equipped with a 60×, 1.4 NA objective and with 405, 488 and 561 nm laser lines to excite DAPI (blu), FITC and TRITC fluorescence signals. Each final confocal image, of 1024 × 1024 pixels and 4096 gray levels, was obtained by maximum intensity projection of 10 optical sections passed through the central region of the cells (recorded at *z*-step size of 300 nm). Volume view with 3D rendering was carried out using the NIS Elements Advanced Research Software (Nikon).

### Western Blot Analysis

Cultured tendon fibroblasts were harvested by scraping. The media were recovered after cell treatment with 0.25 mM L-ascorbic acid for 24 h without FBS, and concentrated with Vivaspin sample concentrators (Vivaspin 2 MWCO10000, GE Healthcare) according to the manufacturer’s operating procedures. Cell lysates and concentrated culture media were resolved by standard SDS–PAGE, electroblotted onto a nitrocellulose membrane (Sardone et al., 2014) and incubated with antibodies against FBN (Sigma), COLI (Abcam), tenomodulin (TNMD; Santa Cruz), actin (Santa Cruz), MMP2 (Santa Cruz), followed by incubation with anti-mouse or anti-rabbit horseradish peroxidize (HRP)-conjugated secondary antibodies. Chemiluminescent detection of proteins was carried out with the ECL detection reagent Kit (GE Healthcare Amersham, Pittsburgh, PA, USA) according to the supplier’s instructions.

### Gelatin Zymography

The activity of MMP-2 and MMP-9 in conditioned medium was detected using gelatin zymography, which was performed under non-reducing conditions in a 7.5% SDS-polyacrylamide gel containing 2 mg/ml gelatin (Mini-PROTEAN II system; Bio-Rad Laboratories Ltd, Hempstead, UK). Gels were washed in 2.5% Triton X-100 to remove SDS and allow renaturation of MMPs, before they were transferred to a solution containing 50 mM Tris (pH 7.5), 5 mM CaCl_2_, and 1 mM ZnCl_2_, followed by incubation at 37°C for 18 h. After staining with Coomassie brilliant blue R250 (Bio-Rad Laboratories, Hercules, CA, USA), pro-MMPs and active MMPs were observed as white lysis bands produced by gelatin degradation.

### Electron Microscopy Study

Tendon fragments were fixed with 2.5% glutaraldehyde in 0.1 M cacodilate buffer, postfixed with 1% osmium tetroxide in 0.1 M cacodilate buffer and embedded in Epon812 epoxy resin following standard procedures. For post embedding immunoelectron microscopy, tendon fragments were fixed with 1% glutaraldehyde in phosphate buffer, embedded in London white resin and ultrathin sections were incubated with an anti-COLVI (Fitzgerald et al., 2008), anti-COLI (Abcam) and anti-FBN (Sigma) as previously reported (Sabatelli et al., 2011), and revealed with anti-rabbit 15 nm colloidal gold conjugated antibody (Sigma). Sections were stained with uranyl acetate and lead citrate and observed with a Jeol JEM-1011 transmission electron microscope operated at 100 kV. For quantitative analysis of COLVI, COLI and FBN immunogold labeling, at least 20 fields for each sample were acquired at the same magnification, and the labeling density was expressed as mean of the number of gold particles/μm^2^ ± SD.

### Statistical Analysis

Statistical analysis was performed by Student’s *t*-test with the Statistical Package for the Social Sciences Software (SPSS, Chicago, IL, USA). The results were considered statistically significant for *p* values less than 0.05.

## Results

**Figure 1:**
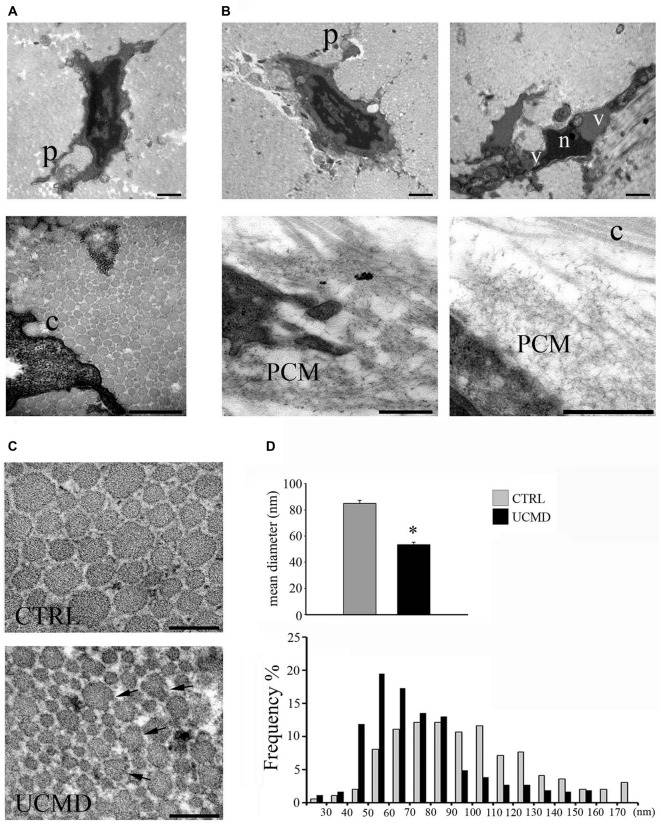
**(A)** Ultrastructural analysis of normal peroneal tendon showing a tenocyte with cellular processes (p, upper panel) which define extracellular matrix compartments containing collagen fibrils. Collagen fibrils are closely associated with the cell membrane (c, lower panel). Scale bar, 1 μm. **(B)** Ullrich congenital muscular dystrophy (UCMD) tenocytes display reduced and irregular cellular processes (p). A necrotic cell with hypercondensed heterochromatin (n) and vacuoles (v) is shown (upper lane, right panel). Note the presence of abnormal microfibrillar material accumulated in the pericellular matrix (PCM) of UCMD fibroblasts. Scale bar, 1 μm. **(C)** Transmission electron microscopy of cross-sectioned normal and UCMD peroneal tendon. Normal tendon displays fibrils with regular profile. In contrast, UCMD tendon displays smaller diameter fibrils; several aberrant fibrils with irregular profile are also observed (arrows). Scale bar, 200 nm. **(D)** Fibril diameter distribution in normal and UCMD tendon. The fibrils diameter distribution was shifted toward smaller diameters in UCMD tendon compared to normal tendon, with a significant difference in mean fibril diameter (± SEM *p < 0.001).

By ultrastructural analysis, normal peroneal tendons showed the presence of scattered tenocytes with long cellular processes and well-packed and oriented collagen fibrils. The tendon matrix appeared mainly constituted by collagen fibrils of different size and few scattered elastin-oxytalan fibers (Figure 1A). Collagen fibrils were present in the pericellular matrix of tenocytes, closely associated with the cell surface (Figure 1A, lower panel).

In contrast, patient’s tenocytes showed reduced cell processes, and occasionally, displayed features of necrotic cells, such as hypercondensed heterochromatin and increased vacuoles (Figure 1B). Numerous tenocytes displayed an abnormal accumulation of microfibrillar/reticular material in the pericellular matrix, which determined the displacement of collagen fibrils from the cell surface (Figure 1B). In addition, cross-sectioned collagen fibrils displayed irregular profiles and a ragged appearance (Figure 1C). The analysis of the fibril diameter distribution revealed a reduced size of patient’s fibrils compared with normal tendon, with an increased number of small fibrils (20–60 nm) and reduced number of large fibrils (>100 nm; Figure 1D); moreover, the mean fibril diameter was significantly reduced (Figure 1D).

**Figure 2:**
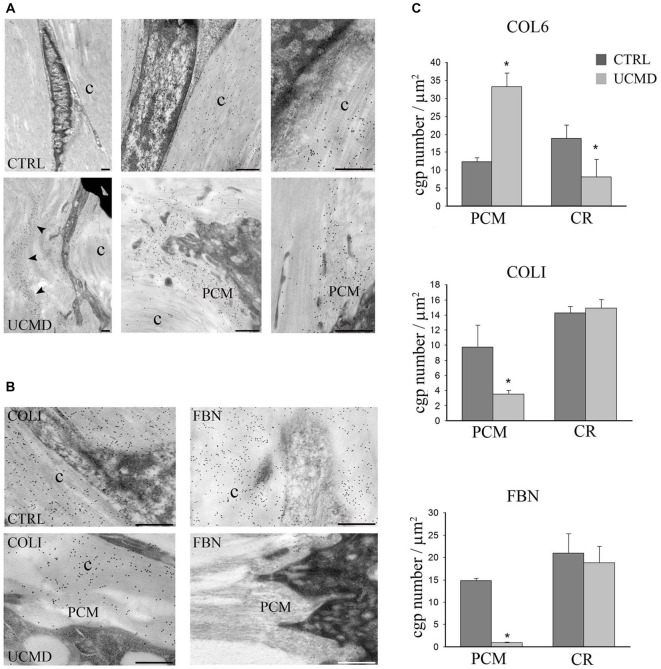
**(A)** Immunoelectron microscopy analysis of collagen VI (COLVI) on tissue sections of normal (upper row) and UCMD (lower row) peroneal tendon sections. In normal tendon, COLVI, identified by 15 nm colloidal gold particles, is detected among collagen fibrils (c). In contrast, in UCMD tendon, COLVI appears concentrated in the pericellular matrix of tenocytes (PCM) and scarcely associated with the collagen fibrils (c). Scale bar, 200 μm. **(B)** Immunoelectron microscopy of collagen I (COLI) (left panels) and fibronectin (FBN; right panels) in normal (upper panels) and UCMD (lower panels), showing a marked reduction of COLI and FBN in the pericellular matrix (PCM) of UCMD tenocytes compared to normal control (c, collagen fibrils). Scale bar, 200 nm. **(C)** Quantitative analysis of the density of colloidal gold particles (cgp) in the pericellular matrix (PCM), and in ECM displaced from the cells (central region, CR) of the UCMD and normal control tendon labeled with anti-COLVI, COLI and FBN antibodies. (± SEM *p < 0.001).

In addition, immunoelectron microscopy study showed that the colloidal gold particles, identifying COLVI, had an uneven distribution in the patient’s matrix tendon, with a conspicuous accumulation in the pericellular matrix of tenocytes and in focal areas of the tendon matrix (Figure 2A); moreover, the association of COLVI with collagen fibrils bundles was apparently impaired compared with the intense labeling detected in normal tendon matrix. In addition, immunoelectron microscopy study of COLI and FBN, both COLVI-related components of tendon matrix, showed a marked reduction in the pericellular matrix of UCMD tendon fibroblasts (Figure 2B). These observations were supported by the quantitative analysis of the number of colloidal gold particles both in the PCM and in areas of tendon matrix displaced from the cells, which further demonstrated significant changes of COLVI, COLI and FBN distribution in the PCM of the UCMD tendon biopsy (Figure 2C).

**Figure 3:**
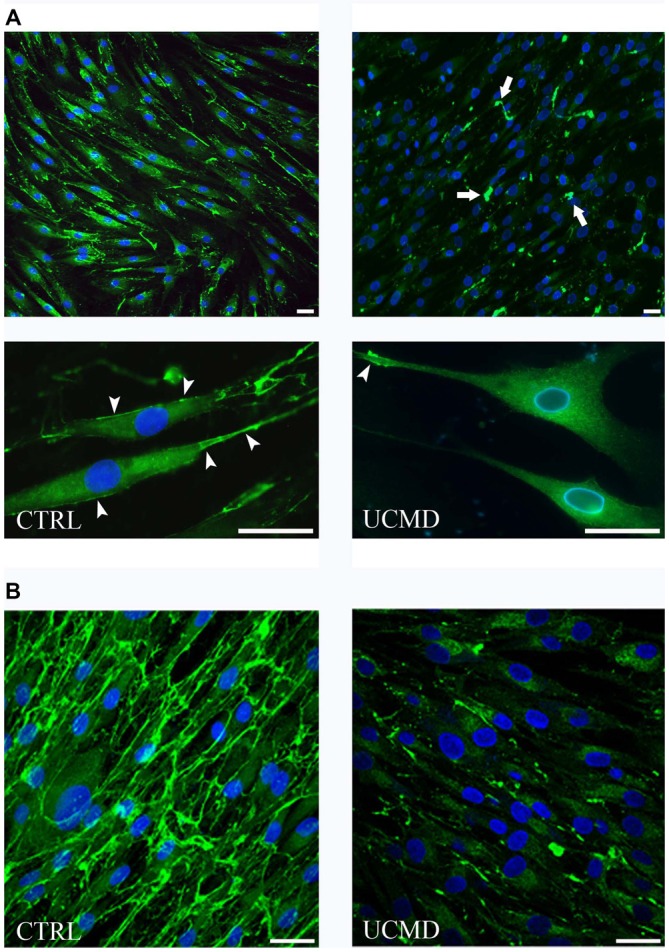
**(A)** Immunofluorescence microscopy of COLVI antibody in normal (CTRL, left panels) and UCMD patient (UCMD, right panels) tenocyte cultures treated with ascorbic acid for 24 h. COLVI is early secreted in normal tenocyte culture and associates with the cell surface (arrowheads). In UCMD tenocyte culture, COLVI is severely reduced in the matrix and form anomalous aggregates (arrows). The association of COLVI with the cell surface is also impaired. Nuclei were stained with DAPI (blue). Scale bar, 20 μm. **(B)** Immunofluorescence microscopy of COLVI in normal (CTRL, left panel) and UCMD (right panel) long term tenocyte cultures showing the abnormalities of COLVI organization in patient sample compared to complex network developed in normal culture. Nuclei were stained with DAPI (blue). Scale bar, 20 μm.

To address further the impact of COLVI deficiency on tendon matrix organization, we studied normal and UCMD patient tendon cultures by immunofluorescence microscopy. Normal and UCMD tenocyte cultures were grown to confluence onto coverslips and treated for different times with ascorbic acid to assess early (24 h) and late (10 days) dynamics of COLVI extracellular assembly. Immunofluorescence microscopy of normal tenocyte cultures showed that COLVI was mainly associated with the cell surface in short term cultures (Figure 3A), while, in long-term samples, it formed a complex and well-developed network (Figure 3B). In UCMD tenocyte culture, COLVI was markedly reduced and poorly associated to the cell surface (Figure 3A); when analyzed in long term cultures, COLVI displayed a spot-like pattern, with large aggregates scattered in the extracellular matrix (Figure 3B).

**Figure 4:**
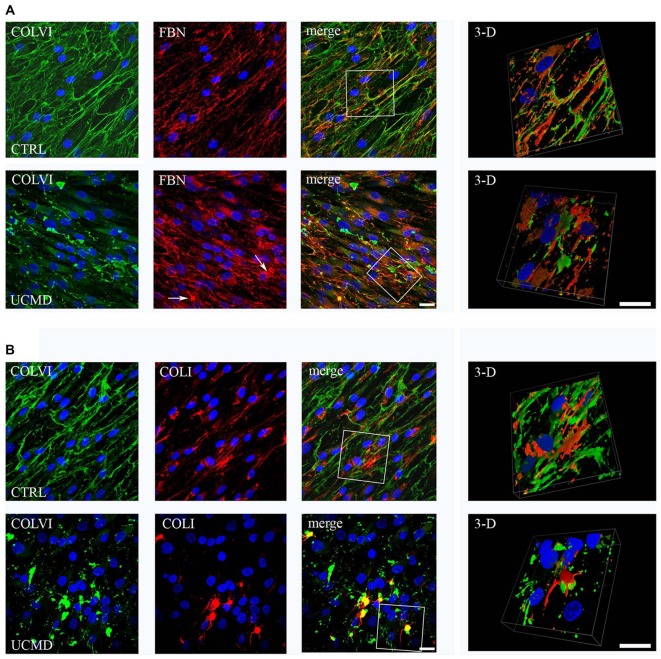
**(A)** Confocal microscopy of (COLVI) and FBN in normal (upper row) and UCMD patient (lower row) tenocyte cultures; 3D surface shaded reconstruction of an enlargement of the area defined by the white box is shown on the right. In normal tendon fibroblast culture, COLVI network co-localizes at discrete site with FBN, as visualized in merge and 3D reconstruction images. In UCMD sample, COLVI forms anomalous aggregates, which also include FBN staining (arrows). Nuclei were stained with DAPI (blue). Scale bar, 50 μm. **(B)** Confocal microscopy of (COLVI) and COLI in normal (upper row) and UCMD patient (lower row) tendon fibroblast cultures; 3D surface shaded reconstruction of an enlargement of the area defined by the white box are shown on the right. In normal tendon fibroblast culture, COLI and COLVI form distinct interconnected networks, as indicated by the partial association visualized in 3D reconstruction. In UCMD tendon culture, COLI forms aggregates that match with COLVI abnormal structures. 3D reconstruction of a particular of the merge image clearly shows that collage I associates with COLVI aggregates. Nuclei were stained with DAPI (blue). Scale bar, 50 μm.

Double labeling of anti-COLVI with anti-FBN revealed moderate changes of the FBN pattern in UCMD tenocyte culture compared to normal control; in fact, small FBN aggregates, which co-localized with COLVI labeling, were detected (Figure 4A). In contrast, COLI organization was severely affected in UCMD culture, as indicated by anomalous aggregates which matched with COLVI deposits (Figure 4B).

**Figure 5:**
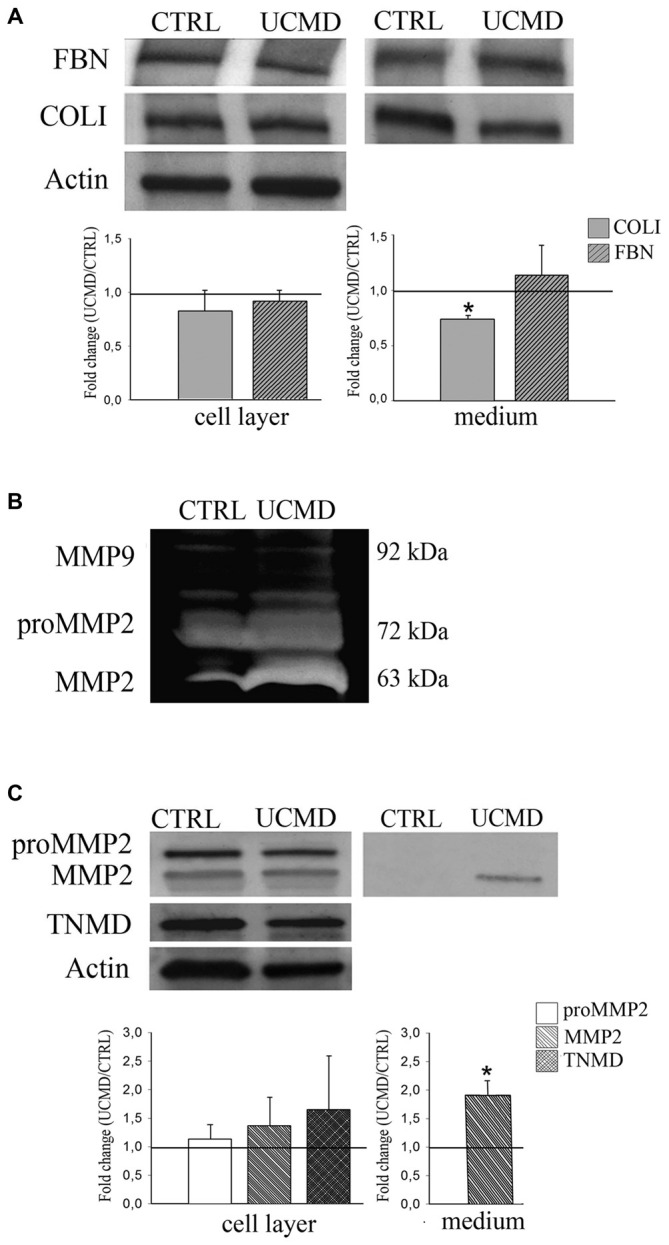
**(A)** Western blot analysis of cell lysate (Cell Layer) and conditioned medium (Medium) of normal (CTRL) and UCMD patient (UCMD) cultured tenocytes of FBN and COLI, and of the relative densitometric quantification; actin was used as a loading control of cell lysates. For cell lysate quantification, protein levels were calculated as relative intensity with respect to actin. The respective protein levels in UCMD patient cells and media were compared to the control (showed by the dark line, set as 1) ± SD (*p < 0.06 vs. control). **(B)** Gelatin zymography of conditioned medium from normal (CTRL) and UCMD patient showing an increased gelatinolitic activity of MMP2 in the culture medium of patient cells. **(C)** Western blot analysis with anti-MMP2 antibody, recognizing both the active (63 kDa) and non-active pro-MMP2 (72 kDa) form in normal (CTRL) and UCMD patient (UCMD) cultured tenocytes (Cell Layer) and conditioned medium (Medium), and the relative densitometric quantification; actin was used as a loading control of cell lysates, while tenomodulin (TNMD), a tenocyte marker, was used to assess the cell phenotype. For cell lysates, protein levels were calculated as relative intensity with respect to actin. The respective protein levels in UCMD patient cells and media were compared to the control (showed by the dark line, set as 1). Error bars indicate SD. (*p < 0.001 vs. control).

Western blot analysis of cell lysates and conditioned medium showed comparable amount of FBN in UCMD and control tenocyte cultures, as indicated by densitometric quantification (Figure 5A); COLI was normally expressed in cell lysate, while it was moderately reduced in the patient conditioned medium (Figure 5A). Furthermore, we investigated the expression and activity of gelatinases MMP2 and MMP9, both involved in tendon matrix turnover (Veidal et al., 2011). Strikingly, gelatin zymography of conditioned medium of patient tendon fibroblasts displayed an increased activity of MMP2, while pro-MMP2 and MMP9 activity were similar to that detected in the control conditioned medium (Figure 5B). Consistent with increased MMP2 gelatinolytic activity, western blot analysis showed an increase of the 63 kDa active form of MMP2 in the conditioned medium of patient tenocytes, while pro-MMP2 was unchanged (Figure 5C). The analysis of MMP2 in cell lysates did not showed differences between UCMD and normal tendon cultures.

## Discussion

Patients with mutations in COLVI genes develop both contractures and distal laxity, possibly due to tendon/ligament involvement. However, the molecular basis and the mechanism leading to these alterations remain unknown. In this article, we report for the first time the impact of COLVI deficiency on the organization of tendon matrix of an UCMD patient, represented by morphological alterations of tendon fibrils, and disruption of tenocyte pericellular matrix organization associated with increased MMP2 activity.

COLVI was markedly reduced both in tendon sections and tenocyte culture of the UCMD patient. This alteration is consistent with the low protein level and the derangement of the COLVI network previously reported in skeletal muscle (Tagliavini et al., 2014) and skin fibroblast cultures of the same patient (Martoni et al., 2009). On the other hand, the majority of COLVI mutations of UCMD patients affect the assembly and secretion of COLVI (Zhang et al., 2002; Merlini and Bernardi, 2008). This may depend on the complex mechanism of COLVI assembly, a multistep process, which involves intracellular formation of dimers and tetramers before secretion (Bruns et al., 1986).

The COLVI quantitative defect was associated to striking alterations of fibrils organization, both in tissue sections and tenocyte cultures. The immunoelectron microscopy analysis of tendon sections showed that COLVI was abnormally accumulated in the pericellular matrix of the patient’s tenocytes and in focal areas of the extracellular matrix, rather than associated to fibrils, as in normal tendon (Bruns et al., 1986). Similarly, aberrant COLVI aggregates were detected in patient’s tenocyte culture, indicating that the low amount of protein secreted is not able to organize a regular matrix. It is interesting to note that in UCMD patients, COLVI may be reduced in the basal lamina surrounding the muscle fibers, but is still present/accumulated in the endomysium and perivascular space (Pan et al., 2003). Aggregates of COLVI have also been reported in the skin (Sabatelli et al., 2011) and in fibroblast cultures of UCMD patients (Hicks et al., 2008; Martoni et al., 2013). It has been proposed that mutant COLVI is not degraded but is aberrantly accumulated in the interstitial space. As possible consequence, COLVI non-functional deposits may hinder the arrangement of extracellular binding partners. Consistent with this hypothesis, the expression of FBN and COLI was severely affected in areas of COLVI accumulation, and in particular in the pericellular matrix of tenocytes, as demonstrated by immunoelectron microscopy on patient’s tendon.

The effect of aberrant COLVI expression on early assembly of COLI and FBN, was better characterized by studying two-dimensional tenocyte cultures. We found that COLI, and to a lesser extent, the FBN organization were affected in patient’s tenocyte cultures. Remarkably, COLI failed to organize a filamentous network aligned to the cell axis, compared to the well-oriented fibrils assembled by normal tenocytes. These data may suggest that collage VI is involved in directional deposition of COLI. It is interesting to note that COLVI in normal cultured tenocytes is associated to the cell surface, and that this pattern was impaired in patient’s culture, pointing to a role of COLVI in cell-surface associated mechanisms, as early steps of collagen fibril assembly (Zhang et al., 2005). The alteration of the FBN pattern in cultured tenocytes is consistent with our previous reports that COLVI deficiency affects the three-dimensional organization of FBN in fibroblast cultures of UCMD and Bethlem myopathy patients (Martoni et al., 2009), and in *Col6A1−/−* null mice (Sabatelli et al., 2001).

The ultrastructural analysis of UCMD tendon showed alterations of fibril morphology and a significant reduction of the number of large fibrils. These data correlated with fibril abnormalities reported in skin of UCMD patients (Kirschner et al., 2005), and in tendons of COLVI myopathy mouse models (Izu et al., 2011; Pan et al., 2013, 2014), and further support the hypothesis of dysfunctional fibrillogenesis.

COLVI interacts with a large number of regulatory molecules, including metalloproteinase MMP-2 (Freise et al., 2009). Interestingly, we found a specific increase of MMP-2 gelatinolytic activity, consistent with the increase of active MMP-2 in the medium of UCMD cultured tenocytes. It is interesting to note that COLVI, and in particular the α2 chain, modulates the activity of MMP-2 by sequestering pro-MMPs in the extracellular matrix, and blocking proteolytic activity (Freise et al., 2009). A moderate increase of MMP-2 activity has been observed in *Col6A1−/−* mice, a COLVI null model (Izu et al., 2011). MMP-2 is involved in the initiation and progression of fibril growth and matrix assembly during tendon development (Jung et al., 2009); increased level of MMP2 has been also reported during tendon healing (Choi et al., 2002). We hypothesize that MMP-2 increased activity may reflect an accelerated tendon matrix turnover in response to defects of COLVI.

Altogether, our data indicate that COLVI deficiency affects both *in vivo* and *in vitro* the organization of matrix tendon, resulting in dysfunctional fibrillogenesis. Fibril alterations have been reported in some forms of Ehlers Danlos Syndrome (EDS) with hypermobile phenotype (Kobayasi, 2004) and in an animal model of EDS with joint phenotype (Sun et al., 2015), suggesting common pathophysiological pathways in this group of connective tissue disorders.

Fibril abnormalities are also regarded as a consequence of decreased loading (Heinemeier and Kjaer, 2011), disuse, and aging sarcopenia (Narici and Maganaris, 2007). Our data, however, point toward a primary tendon dysfunction. In fact, we have shown that alterations of the extracellular matrix were also present in the patient’s cultures, effectively reducing the importance of muscle dysfunction as a determinant of the tendon phenotype.

## Author Contributions

FS performed in culture studies; PS performed the immunogold and the ultrastuctural study; SS performed the confocal analysis; AB, FT, CF, LM and NMM participated in data collection, data interpretation, and reviewed and critiqued the manuscript. All authors listed, have made substantial, direct and intellectual contribution to the work, and approved it for publication.

## Conflict of Interest Statement

The authors declare that the research was conducted in the absence of any commercial or financial relationships that could be construed as a potential conflict of interest.

## References

[B1] Birk D. E. Nurminskaya M. V. Zycband E. I. (1995). Collagen fibrillogenesis in situ: fibril segments undergo post-depositional modifications resulting in linear and lateral growth during matrix development. Dev. Dyn. 202, 229–243. 10.1002/aja.1002020303 7780173

[B2] Bonaldo P. Braghetta P. Zanetti M. Piccolo S. Volpin D. Bressan G. M. (1998). Collagen VI deficiency induces early onset myopathy in the mouse: an animal model for Bethlem myopathy. Hum. Mol. Genet. 7, 2135–2140. 10.1093/hmg/7.13.2135 9817932

[B3] Bruns R. R. Press W. Engvall E. Timpl R. Gross J. (1986). Type VI collagen in extracellular, 100-nm periodic filaments and fibrils: identification by immunoelectron microscopy. J. Cell Biol. 103, 393–404. 10.1083/jcb.103.2.393 3525575 PMC2113834

[B4] Choi H. R. Kondo S. Hirose K. Ishiguro N. Hasegawa Y. Iwata H. (2002). Expression and enzymatic activity of MMP-2 during healing process of the acute supraspinatus tendon tear in rabbits. J. Orthop. Res. 20, 927–933. 10.1016/s0736-0266(02)00016-5 12382955

[B5] Engvall E. Hessle H. Klier G. (1986). Molecular assembly, secretion and matrix deposition of type VI collagen. J. Cell Biol. 102, 703–710. 10.1083/jcb.102.3.703 3456350 PMC2114116

[B6] Fitzgerald J. Rich C. Zhou F. H. Hansen U. (2008). Three novel collagen VI chains, α4(VI), α5(VI) and α6(VI). J. Biol. Chem. 283, 20170–20180. 10.1074/jbc.M710139200 18400749

[B7] Freise C. Erben U. Muche M. Farndale R. Zeitz M. Somasundaram R.. (2009). The α 2 chain of collagen type VI sequesters latent proforms of matrix-metalloproteinases and modulates their activation and activity. Matrix Biol. 28, 480–489. 10.1016/j.matbio.2009.08.001 19698785

[B8] Gara S. K. Grumati P. Urciuolo A. Bonaldo P. Kobbe B. Koch M.. (2008). Three novel collagen VI chains with high homology to the α3 chain. J. Biol. Chem. 283, 10658–10670. 10.1074/jbc.M709540200 18276594

[B9] Heinemeier K. M. Kjaer M. (2011). *In vivo* investigation of tendon responses to mechanical loading. J. Musculoskelet. Neuronal Interact. 11, 115–123. 21625048

[B10] Hicks D. Lampe A. K. Barresi R. Charlton R. Fiorillo C. Bonnemann C. G.. (2008). A refined diagnostic algorithm for Bethlem myopathy. Neurology 70, 1192–1199. 10.1212/01.wnl.0000307749.66438.6d 18378883

[B11] Izu Y. Ansorge H. L. Zhang G. Soslowsky L. J. Bonaldo P. Chu M. L.. (2011). Dysfunctional tendon collagen fibrillogenesis in collagen VI null mice. Matrix Biol. 30, 53–61. 10.1016/j.matbio.2010.10.001 20951202 PMC3778658

[B12] Jung J. C. Wang P. X. Zhang G. Ezura Y. Fini M. E. Birk D. E. (2009). Collagen fibril growth during chicken tendon development: matrix metalloproteinase-2 and its activation. Cell Tissue Res. 336, 79–89. 10.1007/s00441-009-0755-4 19221802 PMC2746393

[B13] Kadler K. E. Holmes D. F. Trotter J. A. Chapman J. A. (1996). Collagen fibril formation. Biochem. J. 316, 1–11. 10.1042/bj3160001 8645190 PMC1217307

[B14] Kirschner J. Hausser I. Zou Y. Schreiber G. Christen H. J. Brown S. C.. (2005). Ullrich congenital muscular dystrophy: connective tissue abnormalities in the skin support overlap with Ehlers-Danlos syndromes. Am. J. Med. Genet. A 132A, 296–301. 10.1002/ajmg.a.30443 15690374

[B15] Knupp C. Pinali C. Munro P. M. Gruber H. E. Sherratt M. J. Baldock C.. (2006). Structural correlation between collagen VI microfibrils and collagen VI banded aggregates. J. Struct. Biol. 154, 312–326. 10.1016/j.jsb.2006.03.023 16713302

[B16] Kobayasi T. (2004). Abnormality of dermal collagen fibrils in Ehlers Danlos syndrome. Anticipation of the abnormality for the inherited hypermobile disorders. Eur. J. Dermatol. 14, 221–229. 15319154

[B17] Koudouna E. Young R. D. Ueno M. Kinoshita S. Quantock A. J. Knupp C. (2014). Three-dimensional architecture of collagen type VI in the human trabecular meshwork. Mol. Vis. 20, 638–648. 24868138 PMC4021673

[B18] Martoni E. Petrini S. Trabanelli C. Sabatelli P. Urciuolo A. Selvatici R.. (2013). Characterization of a rare case of Ullrich congenital muscular dystrophy due to truncating mutations within the *COL6A1* gene C-terminal domain: a case report. BMC Med. Genet. 14:59. 10.1186/1471-2350-14-59 23738969 PMC3681647

[B19] Martoni E. Urciuolo A. Sabatelli P. Fabris M. Bovolenta M. Neri M.. (2009). Identification and characterization of novel collagen VI non-canonical splicing mutations causing Ullrich congenital muscular dystrophy. Hum. Mutat. 30, E662–E672. 10.1002/humu.21022 19309692

[B20] Merlini L. Bernardi P. (2008). Therapy of collagen VI-related myopathies (Bethlem and Ullrich). Neurotherapeutics 5, 613–618. 10.1016/j.nurt.2008.08.004 19019314 PMC4514708

[B21] Narici M. V. Maganaris C. N. (2007). Plasticity of the muscle-tendon complex with disuse and aging. Exerc. Sport Sci. Rev. 35, 126–134. 10.1097/jes.0b013e3180a030ec 17620931

[B22] Nemoto M. Kizaki K. Yamamoto Y. Oonuma T. Hashizume K. (2013). Tenascin-C expression in equine tendon-derived cells during proliferation and migration. J. Equine Sci. 24, 17–24. 10.1294/jes.24.17 24833997 PMC4013982

[B23] Pan T. C. Zhang R. Z. Arita M. Bogdanovich S. Adams S. M. Gara S. K.. (2014). A mouse model for dominant collagen VI disorders: heterozygous deletion of Col6a3 Exon 16. J. Biol. Chem. 289, 10293–10307. 10.1074/jbc.M114.549311 24563484 PMC4036154

[B24] Pan T. C. Zhang R. Z. Markova D. Arita M. Zhang Y. Bogdanovich S.. (2013). COL6A3 protein deficiency in mice leads to muscle and tendon defects similar to human collagen VI congenital muscular dystrophy. J. Biol. Chem. 288, 14320–14331. 10.1074/jbc.M112.433078 23564457 PMC3656288

[B25] Pan T. C. Zhang R. Z. Sudano D. G. Marie S. K. Bönnemann C. G. Chu M. L. (2003). New molecular mechanism for Ullrich congenital muscular dystrophy: a heterozygous in-frame deletion in the *COL6A1* gene causes a severe phenotype. Am. J. Hum. Genet. 73, 355–369. 10.1086/377107 12840783 PMC1180372

[B26] Ritty T. M. Roth R. Heuser J. E. (2003). Tendon cell array isolation reveals a previously unknown fibrillin-2-containing macromolecular assembly. Structure 11, 1179–1188. 10.1016/s0969-2126(03)00181-3 12962636

[B27] Sabatelli P. Bonaldo P. Lattanzi G. Braghetta P. Bergamin N. Capanni C.. (2001). Collagen VI deficiency affects the organization of fibronectin in the extracellular matrix of cultured fibroblasts. Matrix Biol. 20, 475–486. 10.1016/s0945-053x(01)00160-3 11691587

[B28] Sabatelli P. Gara S. K. Grumati P. Urciuolo A. Gualandi F. Curci R.. (2011). Expression of the collagen VI α5 and α6 chains in normal human skin and in skin of patients with collagen VI-related myopathies. J. Invest. Dermatol. 131, 99–107. 10.1038/jid.2010.284 20882040

[B29] Sabatelli P. Gualandi F. Gara S. K. Grumati P. Zamparelli A. Martoni E.. (2012). Expression of collagen VI α5 and α6 chains in human muscle and in Duchenne muscular dystrophy-related muscle fibrosis. Matrix Biol. 31, 187–196. 10.1016/j.matbio.2011.12.003 22226732 PMC3315014

[B30] Sardone F. Traina F. Tagliavini F. Pellegrini C. Merlini L. Squarzoni S.. (2014). Effect of mechanical strain on the collagen VI pericellular matrix in anterior cruciate ligament fibroblasts. J. Cell. Physiol. 229, 878–886. 10.1002/jcp.24518 24356950

[B31] Screen H. R. Berk D. E. Kadler K. E. Ramirez F. Young M. F. (2015). Tendon functional extracellular matrix. J. Orthop. Res. 33, 793–799. 10.1002/jor.22818 25640030 PMC4507431

[B32] Steffen F. Bilzer T. Brands J. Golini L. Jagannathan V. Wiedmer M.. (2015). A nonsense variant in COL6A1 in landseer dogs with muscular dystrophy. G3 (Bethesda) 5, 2611–2617. 10.1534/g3.115.021923 26438297 PMC4683634

[B33] Sun M. Connizzo B. K. Adams S. M. Freedman B. R. Wenstrup R. J. Soslowsky L. J.. (2015). Targeted deletion of collagen V in tendons and ligaments results in a classic Ehlers-Danlos syndrome joint phenotype. Am. J. Pathol. 185, 1436–1447. 10.1016/j.ajpath.2015.01.031 25797646 PMC4419209

[B34] Tagliavini F. Pellegrini C. Sardone F. Squarzoni S. Paulsson M. Wagener R.. (2014). Defective collagen VI α6 chain expression in the skeletal muscle of patients with collagen VI-related myopathies. Biochim. Biophys. Acta 1842, 1604–1612. 10.1016/j.bbadis.2014.05.033 24907562 PMC4316388

[B35] Telfer W. R. Busta A. S. Bonnemann C. G. Feldman E. L. Dowling J. J. (2010). Zebrafish models of collagen VI-related myopathies. Hum. Mol. Genet. 19, 2433–2444. 10.1093/hmg/ddq126 20338942 PMC2876888

[B36] Thakkar D. Grant T. M. Hakimi O. Carr A. J. (2014). Distribution and expression of type VI collagen and elastic fibers in human rotator cuff tendon tears. Connect. Tissue Res. 55, 397–402. 10.3109/03008207.2014.959119 25166893

[B37] Veidal S. S. Karsdal M. A. Vassiliadis E. Nawrocki A. Larsen M. R. Nguyen Q. H.. (2011). MMP mediated degradation of type VI collagen is highly associated with liver fibrosis—identification and validation of a novel biochemical marker assay. PLoS One 6:e24753. 10.1371/journal.pone.0024753 21935455 PMC3173456

[B38] von der Mark H. Aumailley M. Wick G. Fleischmajer R. Timpl R. (1984). Immunochemistry, genuine size and tissue localization of collagen VI. Eur. J. Biochem. 142, 493–502. 10.1111/j.1432-1033.1984.tb08313.x 6432530

[B39] Wiberg C. Heinegård D. Wenglén C. Timpl R. Mörgelin M. (2002). Biglycan organizes collagen VI into hexagonal-like networks resembling tissue structures. J. Biol. Chem. 277, 49120–49126. 10.1074/jbc.M206891200 12354766

[B40] Young B. B. Zhang G. Koch M. Birk D. E. (2002). The roles of types XII and XIV collagen in fibrillogenesis and matrix assembly in the developing cornea. J. Cell Biochem. 87, 208–220. 10.1002/jcb.10290 12244573

[B42] Zhang R. Z. Sabatelli P. Pan T. C. Squarzoni S. Mattioli E. Bertini E.. (2002). Effects on collagen VI mRNA stability and microfibrillar assembly of three COL6A2 mutations in two families with Ullrich congenital muscular dystrophy. J. Biol. Chem. 277, 43557–43564. 10.1074/jbc.M207696200 12218063

[B41] Zhang G. Young B. B. Ezura Y. Favata M. Soslowsky L. J. Chakravarti S.. (2005). Development of tendon structure and function: regulation of collagen fibrillogenesis. J. Musculoskelet. Neuronal Interact. 5, 5–21. 15788867

[B43] Zulian A. Rizzo E. Schiavone M. Palma E. Tagliavini F. Blaauw B.. (2014). NIM811, a cyclophilin inhibitor without immunosuppressive activity, is beneficial in collagen VI congenital muscular dystrophy models. Hum. Mol. Genet. 23, 5353–5363. 10.1093/hmg/ddu254 24852368

